# Nursing Professionals' Awareness of Adverse Drug Reactions and Pharmacovigilance in an Institute of National Importance in India: A Cross-Sectional Study

**DOI:** 10.7759/cureus.49264

**Published:** 2023-11-22

**Authors:** Mangesh Bankar, Sachchidanand Tewari, Subodh Kumar

**Affiliations:** 1 Department of Pharmacology, All India Institute of Medical Sciences, Raebareli, Raebareli, IND; 2 Department of Pharmacology, All India Institute of Medical Sciences, Deoghar, Deoghar, IND

**Keywords:** awareness, practices nursing, knowledge, pharmacovigilance, adverse drug reaction reporting

## Abstract

Background

Globally, there is a growing concern about adverse drug reactions (ADRs) as they can lead to increased hospital admissions and healthcare expenses, lower patient satisfaction with treatment outcomes, and even fatalities. Pharmacovigilance is crucial for minimizing the risks associated with drug therapy, but underreporting of ADRs is a prevalent issue. Nursing professionals are an important stakeholder in ADR reporting, as they are often the first point of contact for patients to identify and report adverse drug reactions.

Objectives

The objectives of the study were to evaluate the knowledge and practices of nursing professionals regarding ADR reporting in a tertiary care teaching institute and the factors influencing their knowledge of ADR reporting.

Methodology

This was a cross-sectional study involving 275 nursing officers at AIIMS Raebareli, who gave their informed consent and completed a questionnaire on demographics, knowledge, and practice domains. Multiple linear regression analysis was used to compare independent variables' influences on knowledge scores. SPSS version 26 (IBM Corp., Armonk, NY, USA) was used for statistical analysis.

Results

The study revealed that the mean knowledge score was 6.378 (total score of 13), with a standard deviation of 2.299 (95% CI 6.10-6.65). About 50.18% of the participants had a knowledge score below 6.5. Multiple regression analysis revealed that working experience, female gender, working in an emergency department, and previous training on ADR reporting significantly influenced the knowledge scores.

Conclusion

The study found that nursing professionals had limited awareness about ADR reporting, even though they worked at an Institute of National Importance. Based on the findings, it can be concluded that there is a need for improved education and training on ADR reporting and to address barriers to reporting, such as a lack of awareness about reporting procedures, and alleviate the fear of legal consequences.

## Introduction

An increased incidence of adverse drug reactions (ADRs) is a growing concern across the world as it can lead to increased hospital admissions, prolonged hospital stays, increased healthcare costs, decreased patient satisfaction with their treatment outcome, and even death [1]. Previous research has shown that ADRs account for 5%-10% of all hospital admissions, prolong the duration of hospital stay by 9%, and increase the cost of treatment by 20% [2].

Pharmacovigilance is crucial in ensuring patient safety and minimising the risks associated with drug therapy [3]. By collecting and analysing data on ADRs, healthcare professionals can make informed decisions about the use of medications and improve patient outcomes. However, the underreporting of adverse drug reactions is a common problem in pharmacovigilance which may be due to a lack of awareness among healthcare professionals about the importance of reporting, fear of legal or professional consequences, and the perception that reporting is time-consuming and burdensome. It is crucial to address these barriers to improve the reporting of adverse drug reactions and thereby patient safety [4,5].

Nursing professionals are an important stakeholder in ADR reporting, as they are often the first point of contact for patients to identify and report adverse drug reactions. However, previous studies conducted in India have shown that the contribution of nursing professionals was very low in adverse drug reaction reporting. Also, their knowledge about ADR reporting was found to be deficient [6-9]. Assessment of their current knowledge, attitude, and practices is crucial to identifying areas where improvement is needed and developing effective strategies for improving ADR reporting, which is especially important for an institute of national importance that should set a standard in ADR reporting for other institutions to follow. Hence, this study was conducted to assess the knowledge and practices of nursing professionals about ADR reporting in a tertiary care teaching institute.

Objectives

The primary objective was to assess nursing professionals' knowledge and barriers to pharmacovigilance practice, and the secondary objective was to identify the factors influencing their awareness regarding ADR reporting.

## Materials and methods

Methodology

Study Design

This was a questionnaire-based cross-sectional study conducted at the All India Institute of Medical Sciences (AIIMS), Raebareli, which is an autonomous Institute under the Central Government of India.

Study Setting

I. Study participants: The study population consisted of nursing professionals working in various departments of the institute.

II. Inclusion and exclusion criteria: Nursing officers working at AIIMS Raebareli who gave voluntary informed consent to participate in the study, irrespective of age, gender, education, and experience, were included. Exclusion criteria included those who did not provide voluntary informed consent to participate in the study. Nursing students studying at AIIMS, Raebareli, were also excluded from the study.

III. Sample size determination: The sample size was determined based on the total number of nursing professionals working at AIIMS, Raebareli. The sample size was calculated using the single proportion formula [10] as follows:

n = N/(1+N*(MOE)2)

N: Total number of nursing officers working in our institute (510)

MOE: Margin of Error (0.05)

n: Desired sample size

Considering a total of 510 nursing professionals were working at the time of participant recruitment, using the above formula, a sample size of 220 nursing professionals was calculated. However, to account for potential dropouts and ensure a more robust sample size, a total of 275 participants were included in the study.

Data Collection Instrument and Procedure

A questionnaire was developed after a thorough literature review and expert consultation to ensure its validity and reliability. The questionnaire consisted of three sections and included demographic details such as age, gender, education, working experience, etc., and two sections comprising 19 questions, of which 13 were about knowledge and six were about barriers to practicing pharmacovigilance. Initially, pilot testing of the questionnaire was done to ensure its reliability and identify any potential issues or areas for improvement. This process involved administering the questionnaire to a small group (25) of participants before distributing it to a larger sample size. The feedback received from the pilot testing helped refine the questionnaire and enhance its effectiveness in gathering data on knowledge and barriers to practicing pharmacovigilance. The results of the pilot testing were not part of the main analysis. After finding out the internal consistency of the questionnaire, it was distributed to the study participants.

The questionnaire had an overall good reliability with a Cronbach's alpha coefficient of 0.86 (95% Confidence Interval [CI] = 075-0.93), indicating a high level of internal consistency among the items. Also, the knowledge subscale had acceptable reliability with Cronbach's alpha = 0.74 (95% CI = 0.54-0.86). Similarly, the barriers to pharmacovigilance questionnaire had good reliability, with Cronbach's alpha coefficient of 0.88 (95% CI = 0.78-0.94). These findings suggested that the questionnaire used in this study was a reliable tool for measuring both overall knowledge and barriers to pharmacovigilance. Additionally, a content validity analysis was conducted to ensure that the questionnaire accurately measures the intended constructs. The content validity of the questionnaire was assessed by a panel of six experts in the field who reviewed the questions and provided feedback on their relevance and clarity.

The answers to the knowledge questionnaire were graded using a scoring system that gives points for responses (yes = 1, no = 0, not sure = 0). The maximum possible score was 13 for the knowledge domain. The participants' knowledge levels were categorized based on their scores, with those below 50% considered to have inadequate knowledge, scores between 50% and 79% considered moderate, and scores of 80% or above indicating good knowledge. This categorization allowed for a clear understanding of the participants' overall knowledge levels in the study.

Statistical analysis

The information was entered into a Microsoft Excel spreadsheet (Microsoft® Corp., Redmond, WA, USA). Descriptive statistics like mean ± standard deviation (SD) were used for quantitative variables and frequency (percentages) for describing the qualitative data. The multiple regression analysis was performed to assess the relationship between the questionnaire score of the knowledge domain and various predictor variables, including age, gender, working experience, working place, previous training and experience of nursing ADR patients, and educational qualification. The assumptions of multiple regression analysis were checked, including normality, linearity, and homoscedasticity. The significance level was set at p < 0.05 to determine statistical significance in the regression analysis. The statistical analysis was performed using SPSS software version 26.0 (IBM Corp., Armonk, NY, USA).

Ethical considerations

The study was approved by the Institutional Ethics Committee, Raebareli. Informed consent was obtained from all participants prior to their inclusion in the study (IEC approval no. F. 3/BIOETHICS/AIIMS-RBL/APPRO/IM/2021/2023-5/11; Study protocol no. 2023-21-IMP-EXP-5). Confidentiality and privacy of the participants' information were maintained throughout the research process. The study was done in accordance with the National Ethical Guidelines for Biomedical and Health Research involving Human Participants (2017) published by the Indian Council of Medical Research.

## Results

Sociodemographic characteristics of the participants

The demographic information collected for this study included age, gender, education level, marital status, working place, previous training on ADR reporting, and experience handling an ADR patient (Tables 1, 2). A total of 275 nursing officers participated in the study having a mean age of 27.18 years and a standard deviation of 2.08 years. The age range of 24-26 years had the most participants (57%), and <24 years had the least (4%). The majority of participants were female (74%) compared to male participants (26%). In terms of education level, the majority (87%) of participants had a bachelor of science (B.Sc.) nursing degree, followed by those with a diploma in nursing (8%) and with a master's degree in nursing (5%). The majority of participants (64%) were unmarried. The mean working experience was 2 years (95% CI: 1.842-2.225).

**Table 1:** Characteristics of the Participants BSc: Bachelor of Science, GNM: General Nursery & Midwifery, MSc: Master of Science, ICU: Intensive Care Unit, OT: Operation Theater, ADR: Adverse Drug Reaction

Variable	Level	Count	Proportion
Gender	Female	203	0.738
	Male	72	0.262
Age (years)	<24	13	0.047
	24-26	157	0.57
	27-30	90	0.327
	>30	15	0.054
Education	BSc	240	0.873
	GNM	22	0.080
	MSc	13	0.047
Marital Status	Married	99	0.360
	Unmarried	176	0.640
Working place	ICU	37	0.135
	Medical	34	0.124
	OT	5	0.018
	Other	151	0.549
	Paediatric	16	0.058
	Surgical	32	0.116
Nursed Patient of ADR?	NO	67	0.246
	YES	208	0.756
Received training on ADR reporting?	NO	264	0.96
	YES	11	0.04

**Table 2:** Descriptive statistics for quantitative variables C.I.: Confidence Interval

Description	Age (years)	Working Experience (years)	Total Knowledge score (max. 13)
Valid	275	251	275
Missing	0	24	0
Mean	27.182	2.033	6.378
95% CI Mean Upper	27.427	2.225	6.650
95% CI Mean Lower	26.936	1.842	6.106
Std. Deviation	2.078	1.549	2.299
Minimum	23.000	0.250	0.000
Maximum	39.000	9.000	13.000
25th percentile	26.000	1.000	5.000
50th percentile	27.000	1.500	7.000
75th percentile	28.500	2.000	8.000

In terms of working place, the participants were spread across different sections of the hospital, including the intensive care unit (14%), surgical wards (11%), medical wards (12%), paediatric wards (5%), and other sections (59%). The majority of the participants (75%) had prior experience of nursing a patient with ADR. However, 96% of them had never received any training in ADR reporting.

Knowledge score of the participants

**Figure 1:**
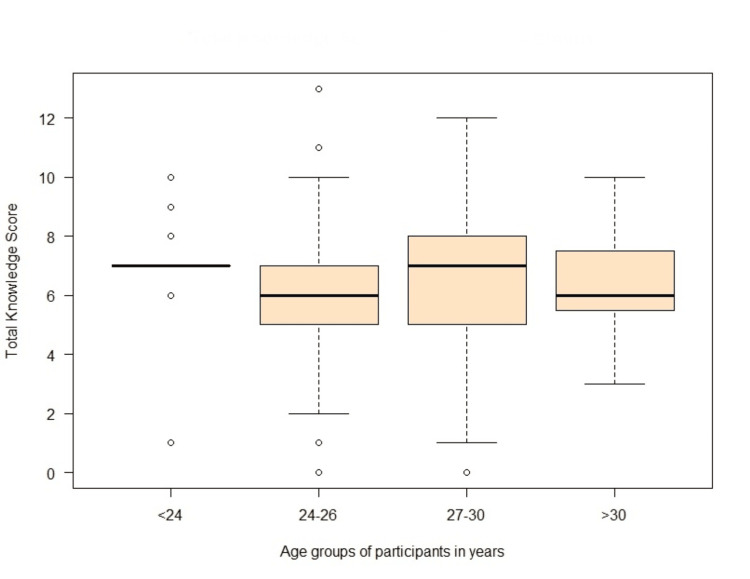
Boxplot of total knowledge score obtained by participants stratified as per age group

**Figure 2:**
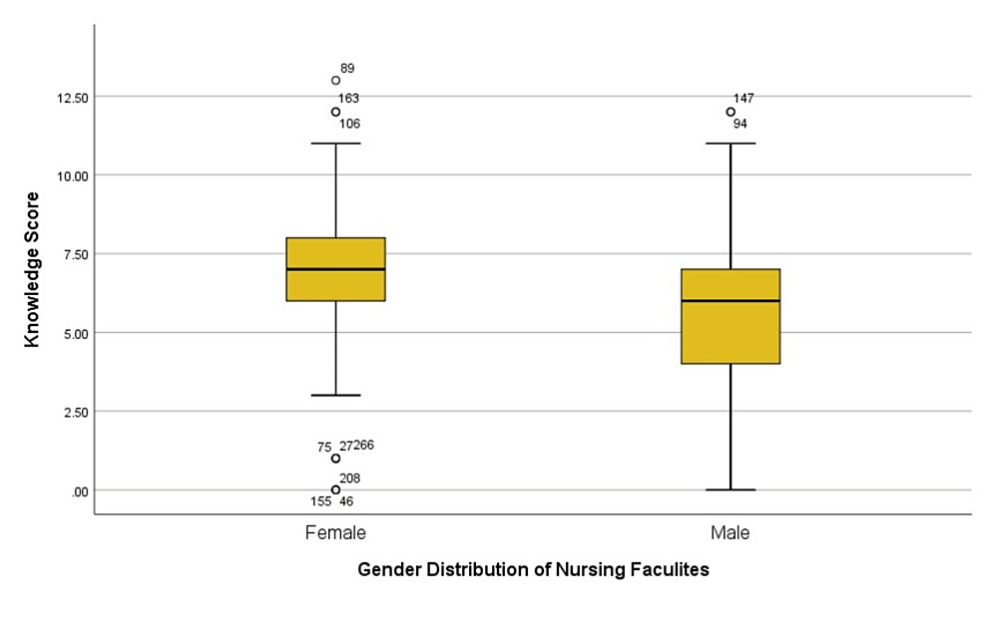
Boxplot showing gender-wise distribution of total knowledge score

The mean knowledge score was 6.378 with a standard deviation of 2.299 (95% CI 6.10-6.65) (Tables 2, 3). Participants in the age groups 27-30 and <24 years received a higher knowledge score than other groups (24-26 years and >30 years) (Figure 1). Females had higher median scores than males (Figure 2). Out of 275 participants, 138 (50.18%) had knowledge scores below 50%, 125 (45%) had moderate knowledge score (50%-79%) whereas only 12 (4.4%) had good knowledge score (>80%).

**Table 3:** Responses of the participants to the knowledge questionnaire. Values expressed as number of responses (proportion). ADRs: Adverse drug reactions

S.N.	Question	Yes	No	Not Sure
1.	ADRs can be reported by any person	154 (0.560)	100 (0.364)	21 (0.076)
2.	All types of ADRs, either serious or nonserious, common or rare types, can be reported?	81 (0.295)	190 (0.691)	4 (0.015)
3.	Do you know the nearest ADR monitoring centre?	11 (0.040)	262 (0.952)	2 (0.007)
4.	Do you know where the national centre for ADR monitoring is located?	30 (0.109)	241 (0.876)	4 (0.015)
5.	Do you know where to obtain the reporting tools for reporting ADRs in your hospital?	50 (0.182)	223 (0.811)	2 (0.007)
6.	Do you know the information that is required on the ADR form?	25 (0.091)	246 (0.895)	4 (0.015)
7.	Do you know where to send the filled ADR form?	92 (0.335)	169 (0.615)	14 (0.051)
8.	To identify safe drugs	207 (0.753)	64 (0.233)	4 (0.015)
9.	To calculate the incidence of ADRs	186 (0.676)	80 (0.291)	9 (0.033)
10.	To identify predisposing factors to ADRs	25 (0.920)	19 (0.069)	3 (0.011)
11.	To identify previously unrecognized ADRs	233 (0.847)	35 (0.127)	7 (0.025)
12.	To serve as an information resource about the characteristics of the ADR	231 (0.840)	38 (0.138)	6 (0.022)
13.	For comparing ADRs of drugs within the same therapeutic class	201 (0.731)	62 (0.225)	12 (0.044)

Barriers to the practice of pharmacovigilance

The majority of participants (77%) who expressed concern that the report might be inaccurate cited this as one of the main reasons for not reporting ADRs. Other barriers mentioned were fear of being accused of wrongly administering the drug (66%), and lack of knowledge about the reporting procedure (55%), while just 14%, 27%, and 24% of participants, respectively, provided reasons for not reporting, such as lack of time owing to a busy workload, uncertainty over the reporting by nursing staff, and non-availability of the reporting form (Table 4).

**Table 4:** Barriers perceived by nursing officers for effective practice of pharmacovigilance ADRs: Adverse drug reactions

S.N.	Concerns expressed by nursing officers	Yes	No	Not Sure
1.	Concern that the report may be wrong	211 (0.767)	56 (0.204)	8 (0.029)
2.	Lack of time and heavy workload	41 (0.149)	230 (0.836)	4 (0.015)
3.	I do not know the reporting procedure	152 (0.553)	117 (0.425)	6 (0.022)
4.	I did not know I was supposed to report ADRs	76 (0.276)	189 (0.687)	10 (0.036)
5.	The reporting form is not available in the hospitals	66 (0.240)	204 (0.742)	5 (0.018)
6.	Fear of being accused of wrongly administering a drug	181 (0.658)	87 (0.316)	7 (0.025)

Multiple regression analysis between knowledge scores and different predictors

We conducted a multiple regression analysis to examine the relationship between the participants' knowledge scores and the predictors: age, gender, years of experience, working place marital status, previous training, and prior experience of nursing an ADR patient (Table 5).

**Table 5:** Linear regression model summary (Dependent variable: Knowledge Score) R: Correlation coefficient; R Square: Proportion of variance in the dependent variable; df1 & df2: Degrees of freedom

Model	R	R Square	Adjusted R Square	Std. Error of the Estimate	Change Statistics				
					R Square Change	F Change	df1	df2	Sig. F Change
1	.605	0.366	0.328	1.884	0.366	9.731	14	236	0.000

Overall, the utility of the multiple regression model was significant in predicting the variance in the knowledge score (R square = 0.366, F (14, 236) = 9.371, p<0.0001). The individual predictors were examined further. The results showed that working experience, female gender, working in the ICU, previous experience of nursing an ADR patient, and prior training were significant positive predictors of the knowledge score of the participants (Table 6).

**Table 6:** Table showing regression coefficients of variables (Dependent Variable: Knowledge Score) ICU: Intensive Care unit; BSc: Bachelor of Science; MSc: Master of Science; ADR: Adverse drug reaction; VIF: Variance inflation factor; B: Coefficients Note: Standardized coefficients and collinearity statistics can only be computed for continuous predictors.

						95% Confidence Interval for B			Collinearity Statistics	
Variables entered	Unstandardized B	Std. Error	Standardized	t	p	Lower	Upper	Tolerance		VIF
(Intercept)	6.278	2.464		2.548	0.011	1.423	11.134			
Age (years)	-0.096	0.075	-0.091	-1.290	0.199	-0.244	0.051	0.732		1.366
Working experience (Years)	0.250	0.093	0.173	2.690	0.008	0.067	0.433	0.732		1.366
Gender (Female)	0.731	0.291		2.510	0.013	0.157	1.305			
Working place (Medical)	-0.147	1.146		-0.128	0.898	-2.404	2.111			
Working place (ICU)	1.176	0.377		3.119	0.002	0.433	1.919			
Working place (Paediatric)	-0.390	1.187		-0.329	0.743	-2.729	1.948			
Working place (Other)	-0.386	1.096		-0.352	0.725	-2.546	1.774			
Working place (Surgical)	0.109	1.147		0.095	0.924	-2.150	2.368			
Education (BSc)	-0.019	0.484		-0.040	0.968	-0.974	0.935			
Education (MSc)	0.010	0.697		0.015	0.988	-1.362	1.383			
Marital Status (Unmarried)	0.051	0.280		0.181	0.856	-0.502	0.604			
Nursed Patient of ADR (YES)	2.166	0.302		7.177	0.001	1.571	2.761			
Received Training (YES)	3.724	0.574		6.486	0.001	2.592	4.855			

## Discussion

The purpose of the study was to evaluate the nursing officers' knowledge of ADR reporting, factors influencing their knowledge of pharmacovigilance, and obstacles to pharmacovigilance practice within an institute of national importance. The study found that, overall, half (50.18%) of nursing officers had a poor level of knowledge regarding ADR reporting. There were certain factors that influenced their knowledge score, such as their years of experience in the field, female gender, working in the ICU, prior training, and previous experience of nursing an ADR patient. Additionally, the study identified several obstacles to pharmacovigilance practice, mainly including a lack of awareness about reporting systems, a fear of reporting wrong information, and a fear of administering the wrong medication to the patient.

In the present study, although the median knowledge score for the younger participants (age groups 24-26 years and <24 years) was higher compared to the older participants (age groups 27-30 and >30 years), age was not found to influence the knowledge score of the participants. Our results are consistent with the previous study conducted in India [11].

The poor knowledge level found in our study is consistent with previous research that has also highlighted gaps in nursing professionals' understanding of pharmacovigilance [12-14]. The reason for this may be due to a lack of emphasis on pharmacovigilance in nursing education programs and limited training opportunities for nurses in this area.

In this study, the majority of the nursing professionals (75%) had prior experience of nursing an ADR patient, which is similar to the findings of previously conducted studies [15,16]. The current study found that prior ADR reporting training and experience managing ADR patients significantly predicted the pharmacovigilance knowledge score. Previous interventional studies evaluating different educational methods to improve ADR reporting among health care professionals (HCPs) suggest a strong improvement in the pharmacovigilance knowledge score of HCPs following an educational intervention [17-19]. This finding highlights the importance of investing in comprehensive training programs for nursing staff, which can significantly improve their understanding and implementation of pharmacovigilance practices. Additionally, incorporating ongoing education and refresher courses can help sustain this knowledge and ensure continued adherence to reporting protocols.

One of the predictors of the knowledge score was found to be the female gender. This result is in line with the earlier research [20]. This might be because, as previous studies have demonstrated, female HCPs show a propensity to report more ADRs than male HCPs, and this has led to greater awareness of ADR reporting [21,22].

Working in the ICU was also found to be a good predictor of ADR-related knowledge in this study. The reason might be due to the increased number of ADRs encountered in ICU patients. The previous review mentioned that there were more adverse drug reactions (ADRs) in ICU patients. This could be attributed to a number of factors, including complex medication regimens, medical complications, and a higher incidence of medication errors in ICU patients [23]. In another review, it is emphasized that serious ADRs have a higher chance of being reported. This could be because healthcare professionals in the ICU may have a heightened awareness of the potential risks associated with medications, leading to increased reporting of serious ADRs [24].

The participants prioritized fear of being accused of prescribing incorrect medication, lack of knowledge about the reporting process, and uncertainty regarding the accuracy of the ADR as reasons for not reporting ADRs. These findings are consistent with previous meta-analysis that has identified similar barriers to ADR reporting among nursing professionals [25]. It is crucial to address these concerns and provide education and support to healthcare professionals in order to improve ADR reporting rates and ensure patient safety.

The strength of the study lies in its use of a large sample size and inclusion of a young population of nursing participants early in their careers, which can be further targeted to improve ADR reporting practices among them. Additionally, the study's focus on nursing professionals specifically allows for targeted interventions to be developed and implemented to address the identified barriers.

Despite the aforementioned strengths of our study, a significant limitation is that it was limited to one institute, which may have limited the generalizability of the results in other healthcare contexts. Therefore, future research should aim to replicate these findings in multiple centers using a more diverse sample of healthcare professionals to enhance the external validity of the study. Additionally, exploring potential strategies to overcome barriers to ADR reporting in different healthcare settings would be valuable in further improving patient safety.

## Conclusions

As expected for an institute of national importance that ought to serve as a model for other healthcare institutions, the study's results revealed that nursing professionals' knowledge was found to be inadequate. This suggests a need for targeted training programs to improve the knowledge and awareness of ADR reporting among nursing professionals in order to ensure patient safety. This can ultimately lead to a culture of increased awareness and proactive reporting of ADRs among nursing professionals, thereby enhancing patient safety in healthcare settings.
